# Glutamate uptake is important for osmoregulation and survival in the rice pathogen *Burkholderia glumae*

**DOI:** 10.1371/journal.pone.0190431

**Published:** 2018-01-02

**Authors:** Yongsung Kang, Ingyu Hwang

**Affiliations:** Department of Agricultural Biotechnology, Seoul National University, Seoul, Republic of Korea; Universite Paris-Sud, FRANCE

## Abstract

Bacteria exhibit an optimal growth rate in culture media with sufficient nutrients at an optimal temperature and pH. In addition, the concentration of solutes plays a critical role in bacterial growth and survival. Glutamate is known to be a major anionic solute involved in osmoregulation and the bacterial cell’s response to changes in solute concentration. To determine how glutamate uptake is involved in osmoregulation in the rice bacterial pathogen *Burkholderia glumae* BGR1, we mutated the *gltI* gene encoding a periplasmic substrate binding protein of a glutamate transport system to abolish glutamate uptake, and monitored the growth of the *gltI* null mutant in Luria-Bertani medium. We found that the *gltI* null mutant showed a slower growth rate than the wild-type strain and experienced hyperosmotic stress resulting in water loss from the cytoplasm in stationary phase. When the incubation time was extended, the mutant population collapsed due to the hyperosmotic stress. The *gltI* null mutant exhibited loss of adaptability under both hypoosmotic and hyperosmotic stresses. The growth rate of the *gltI* null mutant was restored to the level of wild-type growth by exogenous addition of glycine betaine to the culture medium, indicating that glycine betaine is a compatible solute in *B*. *glumae*. These results indicate that glutamate uptake from the environment plays a key role in osmoregulation in *B*. *glumae*.

## Introduction

Bacteria maintain a positive turgor with diverse solutes in water to survive in their niches. Maintaining a positive turgor is essential for cell growth because turgor is generally considered to be the driving force for cell expansion [1, 2]. To respond to osmotic stresses in the environment, bacteria take up or synthesize appropriate solutes called osmolytes [3]. Some osmolytes serve as nutrients for cell growth and contribute to the maintenance of cellular osmolality in bacteria. Osmolytes can accumulate at high concentrations without inhibiting vital cellular processes [4, 5]. Such osmolytes include amino acids (glutamate, glutamine, and proline), amino acid derivatives (betaines, peptides, and *N*-acetylated amino acids), and sugars (trehalose and sucrose) [3, 6].

In enteric bacteria, such as *Escherichia coli* and *Salmonella typhimurium*, the initial response upon osmotic upshift is the accumulation of potassium ions in the cytoplasm of the cells, followed by counterbalance of the potassium charge by the increased uptake and *de novo* synthesis of glutamate [2, 7–9]. Because high cytoplasmic levels of potassium-glutamate can impair enzyme function and alter gene expression [10, 11], the enteric bacteria must replace the potassium-glutamate with other osmolytes called "compatible solutes" that do not affect cellular activities [12]. When glycine betaine is depleted in culture medium, synthesis of trehalose is initiated to balance cellular osmolality in *E*. *coli* [13]. However, non-enteric bacteria have different mechanisms to balance cellular osmolality. It was observed that several Gram-negative bacteria accumulate large amounts of glutamate upon hyperosmotic stress [14]. In *Vibrio harveyi* (formerly *Beneckea harveyi*), the total intracellular amino acid pool was found to increase as the salinity of the medium increased [15]. Glutamate was found to be the predominate amino acid involved in osmoregulation in *V*. *harveyi* [15]. These findings indicate that glutamate is a key player in osmoregulation in Gram-negative bacteria. However, the role of glutamate in osmoregulation has not been characterized in a wide range of Gram-negative bacteria. Furthermore, the role of glutamate transport in osmoregulation has not been completely elucidated.

Many studies on plant pathogenic bacteria suggest that adaptation to osmotic stress is important for survival in soil [16] and for pathogenesis in plants [17, 18]. The plant pathogenic bacteria survive with small amounts of osmolytes in soil. However, the pathogenicity of bacteria is widely influenced by the presence of osmolytes such as betaines, choline, amino acids, and pipecolic acid, which are abundant in plants [19, 20]. We were interested in whether glutamate uptake and cellular osmotic homeostasis are critical for survival of the rice pathogen *Burkholderia glumae*. This bacterium infects rice flowers and causes panicle blight, and disease severity is closely correlated with environmental conditions [21]. We previously demonstrated that the quorum sensing system of *B*. *glumae* negatively controls glutamate uptake and expression of genes involved in the *de novo* synthesis of glutamate, and that quorum sensing-dependent glutamate metabolism is important for bacterial osmolality homeostasis [22]. Thus, we aimed to investigate the physiological roles of the uptake and accumulation of glutamate in the adaptation of *B*. *glumae* cells to osmotic stress. Here, we show that cell growth is retarded in a glutamate-uptake mutant of *B*. *glumae*, and that the inability to take up glutamate from the culture medium causes severe hyperosmotic stress leading to cell death.

## Materials and methods

### Bacterial strains and growth conditions

The bacterial strains and plasmids used in this study are listed in S1 Table. The *B*. *glumae* strains were routinely cultured in Luria-Bertani (LB) medium containing 0.1% (w/v) tryptone, 0.5% (w/v) yeast extract, and 0.5% (w/v) NaCl (USB, Cleveland, OH, USA) at 37°C.

### Generation of the glutamate uptake mutant

**Fig 1:**
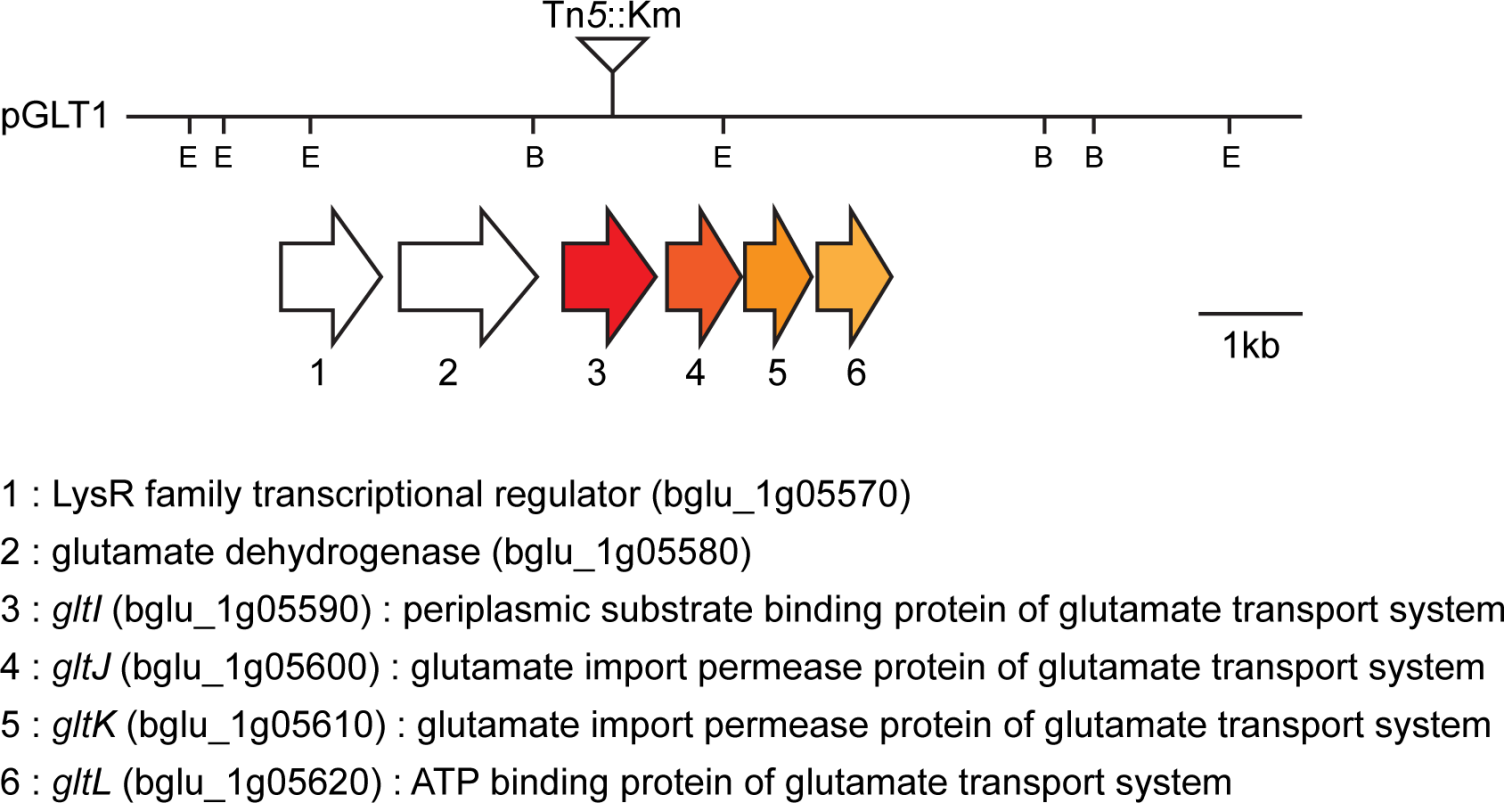
Genetic organization of the *gltI* gene in *B*. *glumae*. Lanes represent the restriction map of pGLT1 plasmid DNA. Colored arrows below the restriction map represent the schematic organization of the *gltI* locus. Vertical bars on the map indicate the positions of Tn*5* insertions. Genetic information and gene identities were obtained from the *B*. *glumae* BGR1 genome database (GenBank accession numbers: CP001503–CP001508). The restriction enzyme sites are indicated as follows: E, *Eco*RI; B, *Bam*HI.

We mutagenized a cosmid clone pGLT1 carrying the *gltI* (locus ID: bglu_1g05590) gene encoding a periplasmic substrate binding protein of the glutamate transport system using EZ-Tn*5*^TM^ <KAN-2> (Epicentre, Madison, WI, USA), as described in the manufacturer’s protocols. Genetic information for gene manipulations was obtained from the *B*. *glumae* BGR1 genome database (GenBank accession numbers: CP001503–CP001508). The mini-Tn5 was inserted at the 3’ end of *gltI*, between 467 and 468 bp, for a total length of 893 bp in pGLT1 (Fig 1). A single Tn*5*::Km insertion was verified by direct sequence analysis using DNA sequencing primers (KAN-2 FP-1/RP-1) as instructed by the manufacturer. To generate BGLT1, the mutagenized plasmid carrying the Tn*5*::Km insertion was first transformed into *E*. *coli* S17-1, introduced into wild-type BGR1 by conjugation, and finally introduced into the chromosome by marker-exchange. All marker exchanges were confirmed with Southern hybridization analysis.

### Measurement of cellular glutamate concentrations

The wild-type *B*. *glumae* BGR1, the *gltI* mutant BGLT1, and the BGLT1(pGLT1) strains were grown in LB broth for 6, 12, 24, and 36 h at 37°C. The bacterial pellets were washed twice with 20 mM Tris-HCl, pH 7.4, and lysed by sonication with a VCX-400 sonicator (Sonics & Materials, Newton, CT, USA). The lysates were cleared by centrifugation at 10,000 × *g* for 20 min. The cellular proteins were precipitated and removed by trichloracetic acid at a final concentration of 10% (w/v). The supernatants were analyzed in a Triple TOF 5600 Q-TOF liquid chromatography tandem mass spectrometry (LC/MS/MS) system (ABSciex, Redwood City, CA, USA) using an Ultimate 3000 RSLC high performance liquid chromatography (HPLC) system (Thermo Fisher Scientific Inc., Waltham, MA, USA), including a degasser, an auto-sampler, Diode array detector, and a binary pump to measure internal glutamate pools. The LC separation was performed on a column with a mobile phase A (0.1% formic acid in water) and a mobile phase B (0.1% formic acid in acetonitrile). The flow rate was 0.25 ml/min. The linear gradient was as follows: The auto-sampler was set at 4°C. The injection volume was 1–5 μl. Mass spectra were acquired under positive electrospray ionization with an ion spray voltage of 4500 V. The source temperature was 450°C. The curtain gas, ion source gas 1, and ion source gas 2 were 35, 65, and 55 psi, respectively. Two full-scan mass spectra were acquired over an *m*/*z* range of 50−2000 on the MS mode. The data were collected using Analyst TF 1.7 software (https://sciex.com/products/software/analyst-tf-software) and analyzed using PeakView 2.2 (https://sciex.com/products/software/peakview-software), MarkerView v.1.2.1.1 (https://sciex.com/support/knowledge-base-articles/markerview-1-3-software-release-notes-1501498023610), and Elements v.1.3.1 (https://sciex.com/applications/life-science-research/metabolomics/discovery-metabolomics).

### Measurement of cellular potassium ion contents

Internal potassium ion fluctuations during growth of *B*. *glumae* strains were measured using an inductively coupled plasma (ICP) atomic emission spectrometer ICP-730ES (Varian, Mulgrave, Australia). Wild-type *B*. *glumae* BGR1, the *gltI* mutant BGLT1, and strain BGLT1(pGLT1) were grown in LB broth for 6, 12, or 24 h at 37°C. The cells were harvested and normalized by weight. The operating conditions for the spectrometer were set to the manufacturer’s specifications (plasma power: 1,500 W; plasma flow: 14 l/min; auxiliary flow: 1.5 l/min; nebulizer flow: 0.7 l/min; pump rate: 15 rpm; and stabilization delay: 30 s).

### Measurement of osmolality

The internal osmolality of the *B*. *glumae* strains was measured as previously described [23]. The wild-type *B*. *glumae* BGR1, the *gltI* mutant BGLT1, and the BGLT1(pGLT1) strains were grown in LB broth for 6, 12, 24, and 36 h at 37°C. At each time point, the cells were harvested from the cultures, the supernatants were removed, and the cells were re-suspended in 0.2 ml of distilled water. An equal weight of each sample was boiled for 10 min at 100°C. The lysates were cleared by centrifugation at 10,000 × *g* for 20 min. The osmolality of the samples was measured with a freezing point osmometer (Osmomat 3000 Basic, Gonotec, Berlin, Germany). The instrument was calibrated using 300 and 1,000 mOsm/kg with sodium chloride solution standards.

### RNA isolation and quantitative reverse transcription polymerase chain reaction (qRT-PCR)

Total RNA was isolated from *B*. *glumae* strains using the RNeasy mini kit (Qiagen, Venlo, Netherlands) according to the manufacturer’s instructions, and the isolated RNA was treated with RNase-free DNase I (Ambion, Austin, TX, USA) to remove genomic DNA. Reverse transcription was performed using 1 μg of total RNA and M-MLV Reverse Transcriptase (Promega, Madison, WI, USA) for 1 h at 42°C. The primer pairs used for qRT-PCR are listed in S2 Table. Amplification of 16S ribosomal RNA (rRNA) served as a positive control. The transcriptional levels were determined using SsoFast EvaGreen Supermix (Bio-Rad, Hercules, CA, USA) and the CFX96 Real-Time PCR System (Bio-Rad). All PCR assays were performed three times, and all data were normalized to the 16S rRNA gene using the Bio-Rad CFX Manager software.

### Transmission electron microscopy (TEM)

The wild-type *B*. *glumae* BGR1, the *gltI* mutant BGLT1, and the BGLT1(pGLT1) strains were grown for 36, 48, and 60 h at 37°C, harvested by centrifugation at 10,000 × *g* for 10 min, fixed with 2.5% glutaraldehyde for 2 h, post-fixed in 1% osmium tetroxide for 2 h, dehydrated in a graded ethanol series (30–100%), and embedded in Spurr’s resin. Ultrathin sections were prepared using an ultramicrotome (EM UC7, Leica, Wetzlar, Germany) and placed on 150-mesh coated copper grids followed by staining with 2% uranyl acetate and lead citrate. The electron micrographs were acquired and recorded using the LIBRA 120 energy filtrating microscope (Carl Zeiss, Oberkochen, Germany) at 100 kV.

### Statistical analysis

All statistical analyses and analysis of variance tests were followed by Tukey’s honest significance difference post hoc analysis using IBM SPSS Statistics software (version 20 x86-x64; IBM corp., Armonk, NY, USA).

## Results

### Growth of the *gltI* mutant

**Fig 2:**
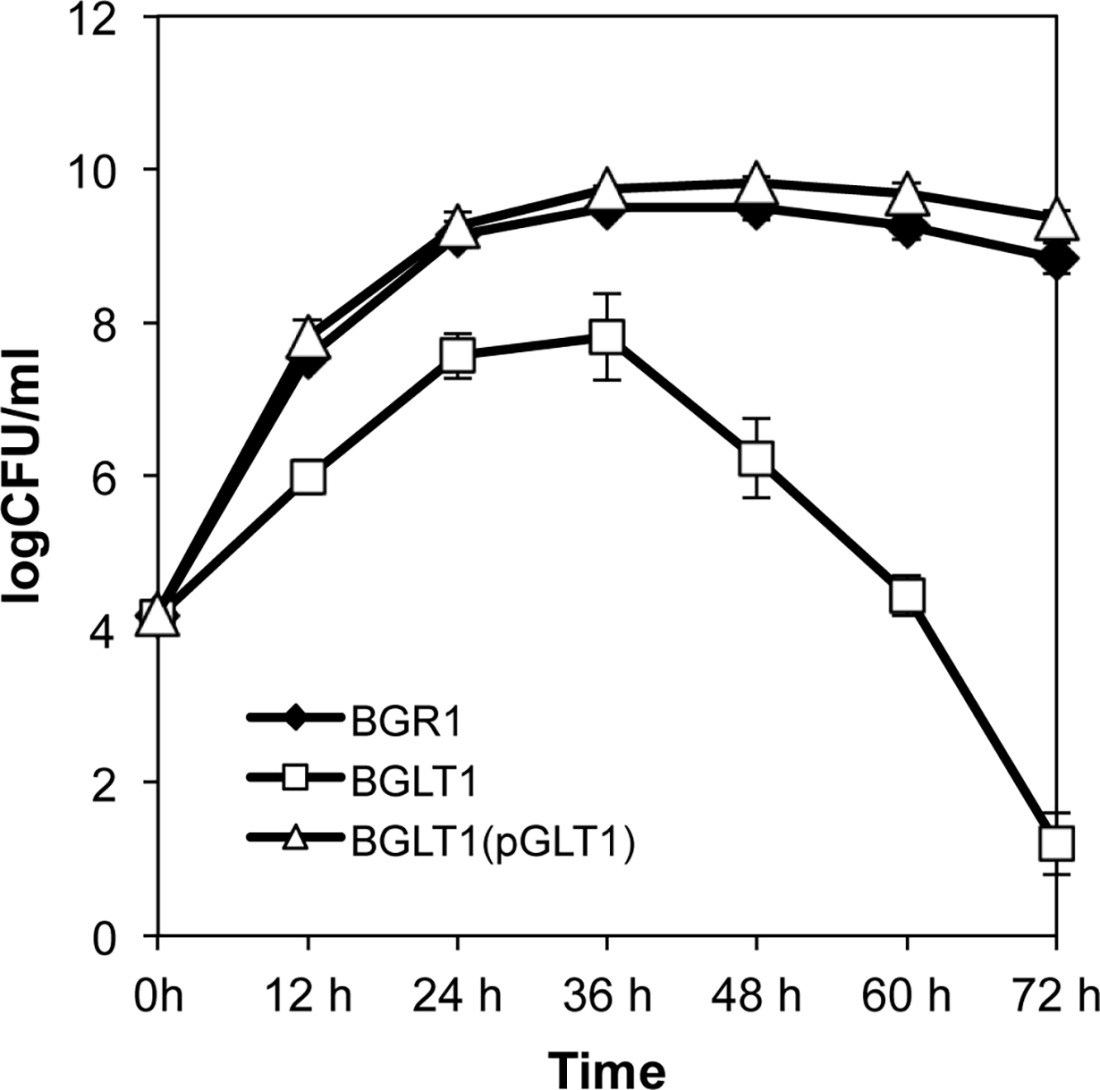
Growth of the *Burkholderia glumae gltI* null mutant in Luria-Bertani (LB) medium. Growth of the *B*. *glumae* wild-type strain BGR1, the *gltI* mutant (BGLT1), and the *gltI* mutant complementation with pGLT1 in LB medium. Error bars indicate the standard error (SE) ranges of three independent experiments.

To investigate whether the uptake of glutamate affects the growth rate of *B*. *glumae* in LB medium, we monitored the growth of wild-type BGR1, the *gltI* mutant BGLT1, and BGLT1(pGLT1) strains. The growth of the *gltI* mutant BGLT1 was significantly retarded at an early exponential stage compared to the wild-type strain, but the growth was recovered by genetic complementation (Fig 2). The *gltI* mutant BGLT1 showed a marked population decline in stationary phase (after 36 h incubation), whereas the wild type and the complemented strain survived in stationary phase without significant population decline in extended incubation periods (Fig 2). These results indicated that the uptake of glutamate is important for bacterial growth and stationary-phase survival in *B*. *glumae*.

### Adaptability of the *gltI* mutant under osmotic stress

**Fig 3:**
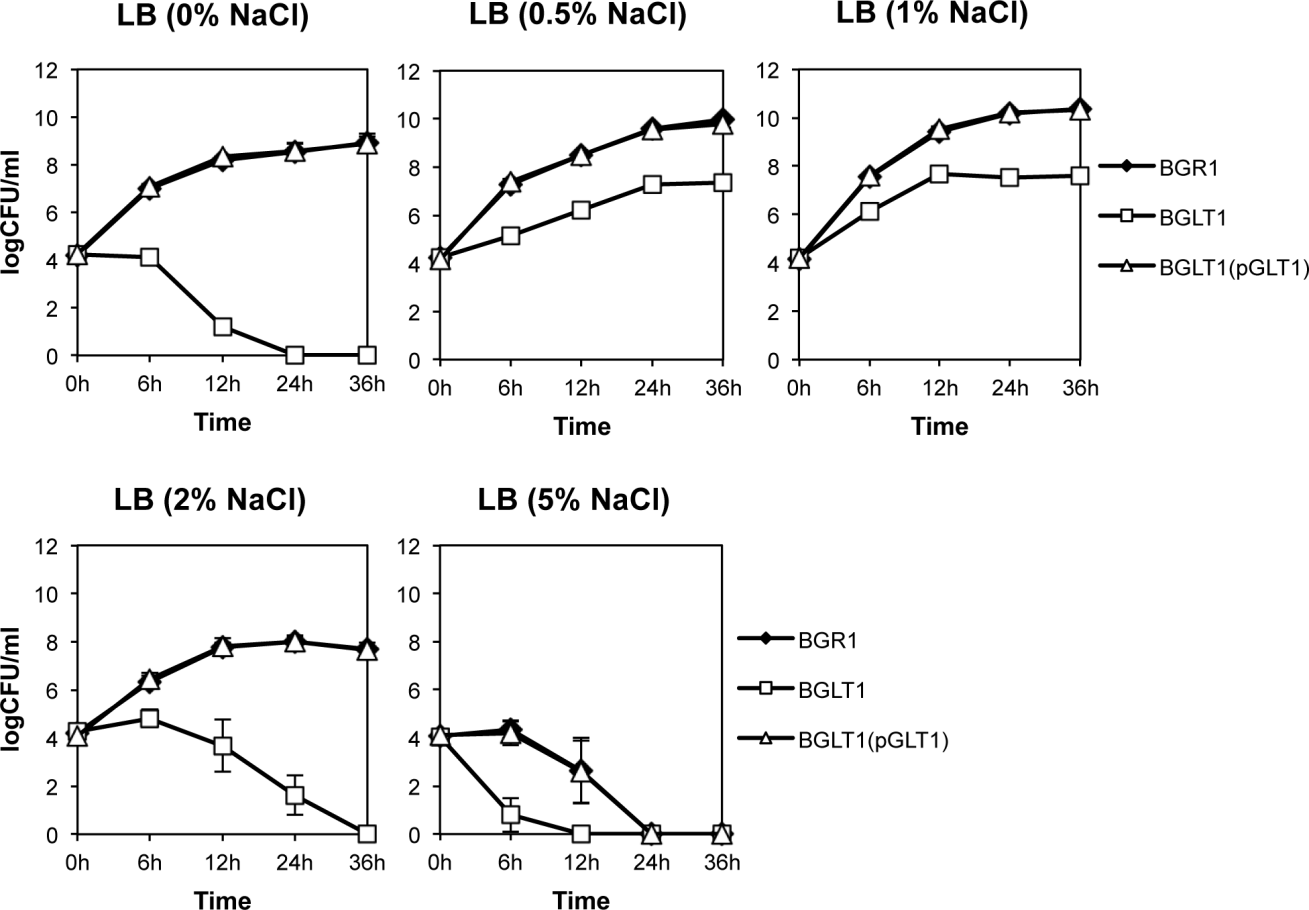
The loss of adaptability in the *gltI* mutant under hypoosmotic and hyperosmotic conditions. The *B*. *glumae* wild-type strain BGR1, the *gltI* mutant (BGLT1), and the *gltI* mutant complementation with pGLT1 were grown in LB medium with various concentrations of NaCl (0–5%). Error bars indicate the SE ranges of three independent experiments.

To determine the physiological roles of the glutamate uptake system of *B*. *glumae* when subjected to osmotic stress, the wild-type strain BGR1, the *gltI* mutant BGLT1, and the complementation strain BGLT1(pBGLT1) were grown in LB broth containing NaCl ranging from 0 to 5%. The wild-type strain BGR1 maintained a maximum population density at approximately 1 × 10^10^ colony forming units per ml (CFU ml^-1^) and below 1 × 10^9^ CFU ml^-1^ in isotonic (1% NaCl), hypotonic (0% NaCl), and hypertonic (2 and 5% NaCl) conditions, respectively (Fig 3). However, there was a significant population decrease in extremely high salt concentration (5% NaCl) (Fig 3). The population of the *gltI* mutant reached less than 1 × 10^8^ CFU ml^-1^ in the isotonic (1% NaCl) condition, rapidly decreased 6 h after incubation, and declined completely after 24−36 h (Fig 3). The population of the complemented strain BGLT1(pGLT1) restored growth comparable to the wild-type strain under the osmotic stress conditions (Fig 3). These results suggest that the glutamate uptake system plays an important role in the adaptability of *B*. *glumae* to unfavorable osmotic conditions.

### Low cellular osmolality of the *gltI* mutant

**Fig 4:**
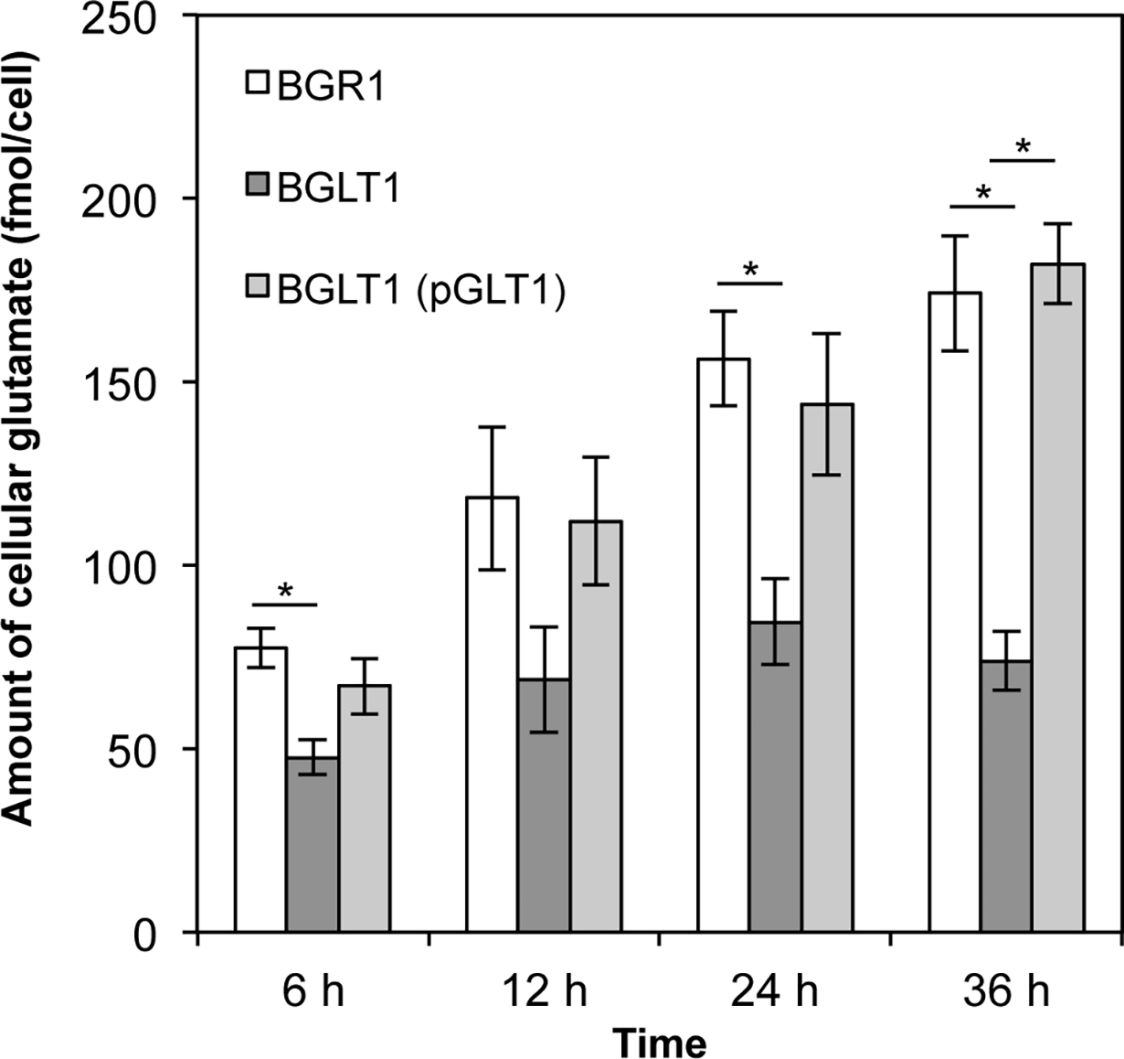
Low cellular glutamate levels in the *gltI* mutant during growth. The intracellular amounts of glutamate in the *B*. *glumae* wild-type strain, *gltI* mutant, and *gltI* mutant complementation during growth. Cells of *B*. *glumae* strains were harvested at each sampling time (6, 12, 24, and 36 h). Glutamate pool sizes were assessed by integration of peaks generated by high performance liquid chromatography with fluorescence detection (HPLC-FLD) and normalized to colony forming units (CFU). Error bars indicate the SE ranges of three independent experiments. The asterisks (*) represent a significant difference (p < 0.05) in glutamate pools between the *gltI* mutant and the wild-type strain or the complement as determined by analysis of variance (ANOVA)/Tukey’s correction for multiple comparisons.

To determine whether the lack of glutamate uptake influences the cellular levels of glutamate in the *gltI* mutant, the cellular glutamate concentration of the three strains (wild type, *gltI* mutant, and its complementation) was analyzed during growth in LB medium. During the exponential growth phase, the cellular glutamate concentration of the wild-type strain increased and the strain maintained a high cellular glutamate concentration during stationary phase (Fig 4). However, the cellular glutamate concentration of the *gltI* mutant increased slowly during the exponential phase and was lower than that of the wild-type strain during all growth periods (Fig 4). The cellular glutamate level of the complemented strain was comparable to the wild-type strain (Fig 4).

**Fig 5:**
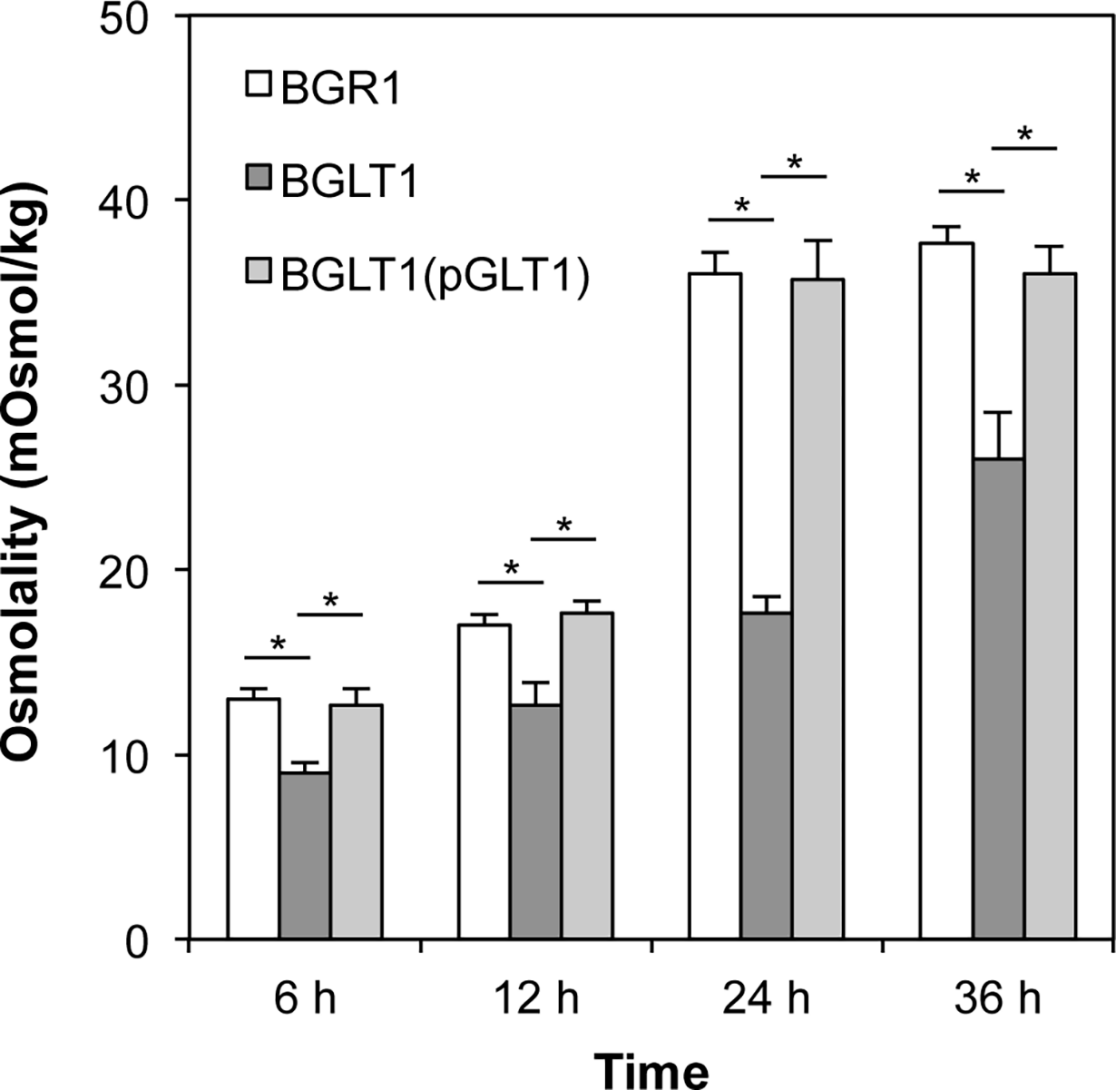
Slow increase in cellular osmolality in the *gltI* mutant. The cellular osmolality levels in the wild-type strain (BGR1), the *gltI* mutant (BGLT1), and *gltI* mutant complementation with pGLT1 in LB medium. The levels of cellular osmolality were measured from *B*. *glumae* strains cultured for 6, 12, 24, and 36 h. All samples were normalized to weight of cells. Error bars indicate the SE ranges of three independent experiments. The asterisks (*) indicate a significant difference (p < 0.05) in osmolality among the *B*. *glumae* strains as determined by ANOVA/Tukey’s correction for multiple comparisons.

To determine if differences in glutamate levels in the wild-type, *gltI* mutant BGLT1, and BGLT1(pBGLT1) strains affected cellular osmolality, we measured the cellular osmolality of each strain during growth. The cellular osmolality of the *gltI* mutant was significantly lower than that of the wild-type strain at all time points (Fig 5). The complemented *gltI* mutant exhibited a similar cellular osmolality to that of the wild-type strain (Fig 5). These results showed a positive correlation between cellular osmolality and glutamate concentration. We also measured cellular potassium ion fluctuations in the *B*. *glumae* strains using ICP atomic emission spectrometry. No differences in internal potassium ion concentrations during growth were detected among the strains (S1 Fig). These results indicate that both the wild-type *B*. *glumae* and the *gltI* mutant maintained an internal pool of potassium ions during growth. This indicates that the *gltl* mutant’s decrease in cellular osmolality is caused by a lack of uptake of external glutamate.

### Expression of genes involved in the *de novo* synthesis of glutamate in the *gltI* mutant

**Fig 6:**
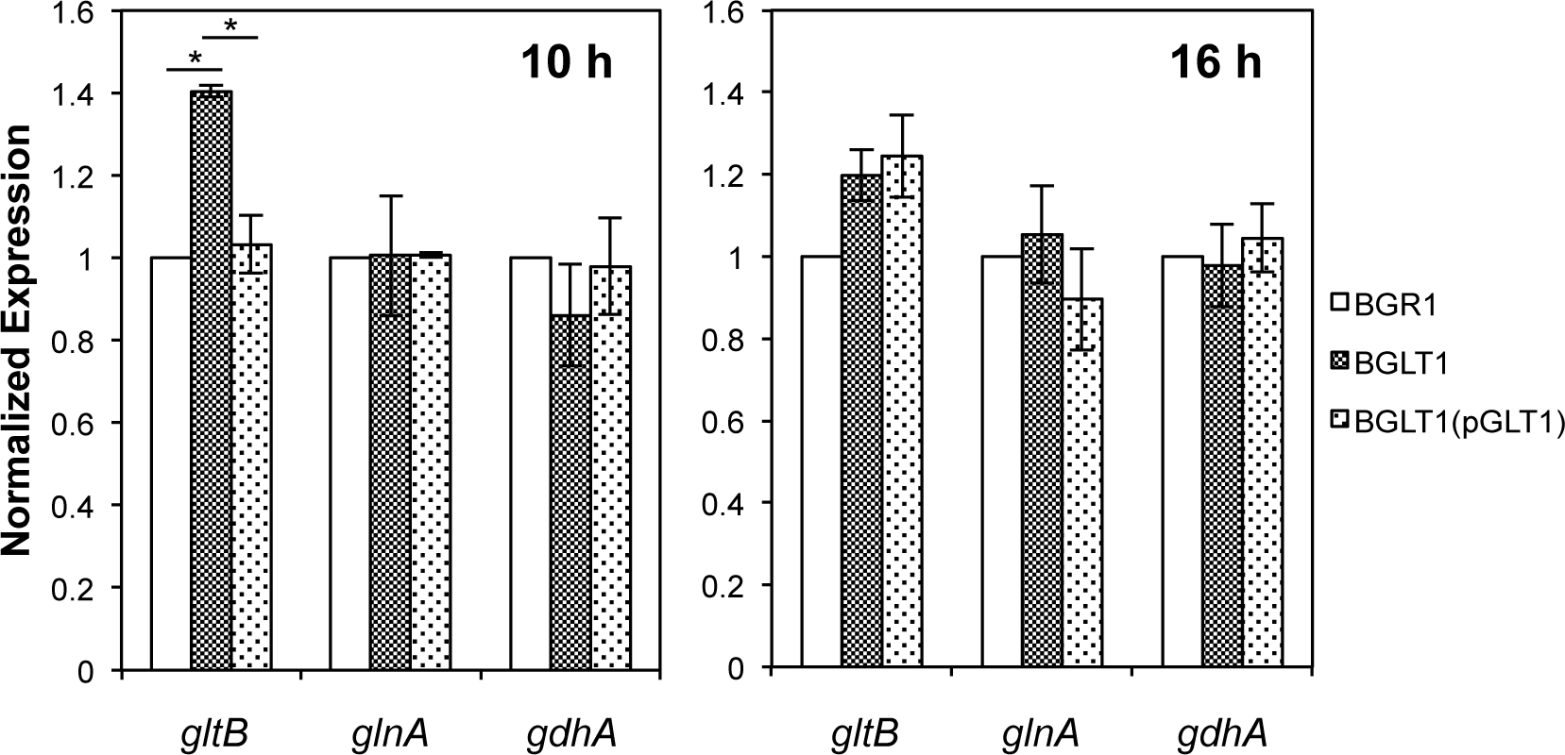
Expression levels of genes involved in the *de novo* synthesis of glutamate in the *B*. *glumae* strains. The gene expression levels of the *gltB*, *glnA*, and *gdhA* genes encoding for glutamine oxoglutarate aminotransferase, glutamine synthetase, and glutamate dehydrogenase, respectively, were quantified in the wild-type strain, *gltI* mutant, and *gltI* mutant complementation after 10 and 16 h of incubation by quantitative reverse transcription polymerase chain reaction (qRT-PCR) with three biological replicates. The bars indicate ± SE. The asterisks (*) represent a significant difference in expression level (p < 0.05) between the *gltI* mutant and the wild-type strain or the complementation as determined by ANOVA/Tukey’s correction for multiple comparisons.

Because glutamate levels could be influenced by genes involved in the *de novo* synthesis of glutamate, including glutamine synthetase (GS), glutamine oxoglutarate aminotransferase (GOGAT), and glutamate dehydrogenase (GDH), we compared the expression levels of the *glnA* (locus ID: bglu_1g25000), *gltB* (locus ID: bglu_1g03130), and *gdhA* (locus ID: bglu_1g05580) genes encoding GS, GOGAT, and GDH, respectively, in the wild-type strain, the *gltI* mutant BGLT1, and the complemented *gltI* mutant BGLT1(pGLT1). The expression of the *gltB* gene, but not the *glnA* and *gdhA* genes, was increased significantly in the *gltI* mutant during exponential growth (10 h) (Fig 6). In stationary phase, however, no significant differences in the expression of the *glnA*, *gltB*, and *gdhA* genes were observed between the wild type and the *gltI* mutant (Fig 6).

### Glycine betaine as a compatible solute in the *gltI* mutant

**Fig 7:**
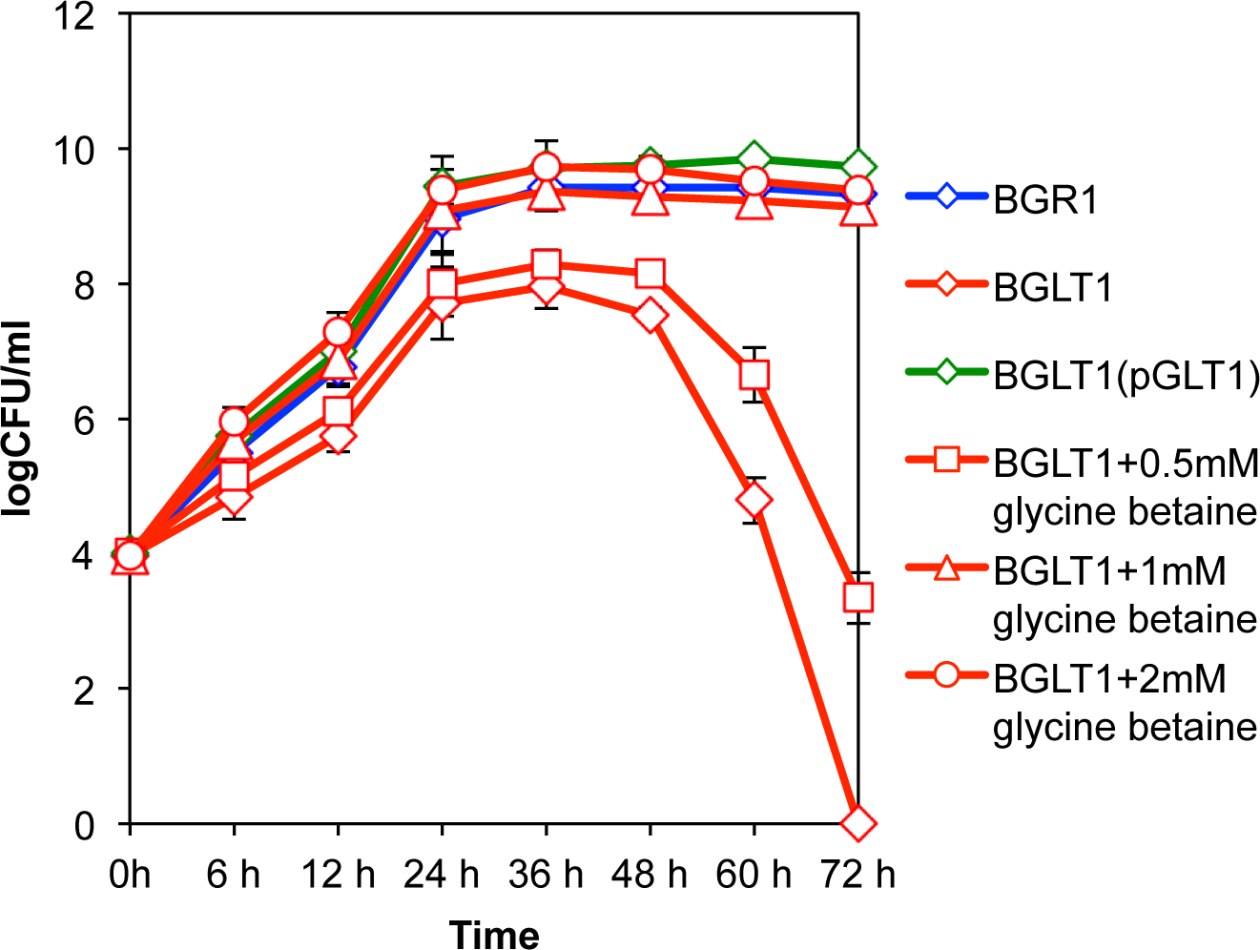
Effect of the addition of glycine betaine on the growth of the *gltI* mutant. Growth of the *B*. *glumae* wild-type strain BGR1, the *gltI* mutant (BGLT1), and *gltI* mutant complementation with pGLT1 [BGRT1(pBGLT1)] with various concentration of glycine betaine as a compatible solute in LB media. Error bars indicate the SE ranges of three independent experiments.

Because the growth of the *gltI* mutant was retarded due to lack of glutamate uptake, we tested whether glycine betaine can function as a compatible solute to recover the growth of the mutant. The exogenous addition of glycine betaine to the culture medium restored the growth of the *gltI* mutant (Fig 7). Addition of 1 mM glycine betaine fully recovered the growth rate of the *gltI* mutant in exponential phase, and no population decline was detected in stationary phase (Fig 7). In the wild-type strain and the complemented *gltI* mutant, an improved growth rate or subsistence on the solute were not observed by exogenous addition of glycine betaine (S2 Fig).

### Cell death of the *gltI* mutant due to hyperosmotic stress

**Fig 8:**
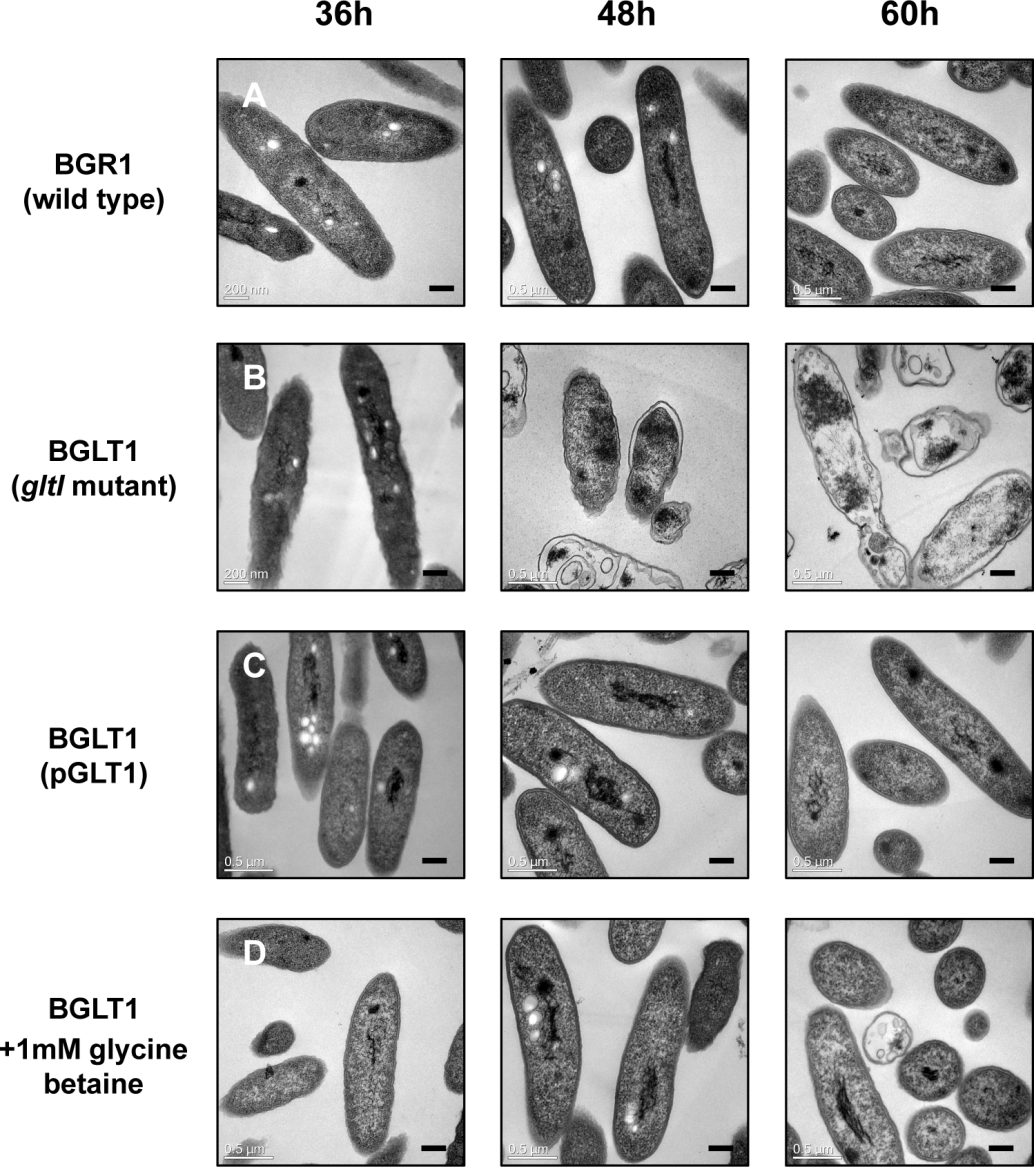
Transmission electron microscopy (TEM) examination of the *gltI* mutant under hyperosmotic stress. TEM ultrathin section micrographs of *B*. *glumae* wild-type strain BGR1, the *gltI* mutant BGLT1, and the *gltI* mutant complementation with pGLT1 [BGRT1(BGLT1)] with exogenous addition of glycine betaine for 36, 48, and 60 h. All micrographs were picked from at least 100 pictures showing similar results. Scale bars indicate 0.2 μm.

To assess the nature of the population decline of the *gltI* mutant due to hyperosmotic stress, we observed ultrathin-sectioned cells using TEM. Intact cell structures with a typical rod-shape were observed in the wild-type strain, whereas the *gltI* mutant exhibited an irregular cell wall shape 36 h after incubation (Fig 8A and 8B). After 48 h, the mutant cells exhibited severe morphological changes, including shrinking of the cytoplasm and separation of the inner cell membrane from the cell wall, indicating that the *gltI* mutant cells experienced hyperosmotic stress as a result of internal water loss (Fig 8B). The cytoplasm of most mutant cells was not stained with 2% uranyl acetate and lead citrate, which was indicative of less dense cytoplasm 60 h after incubation (Fig 8B). These morphological changes in the *gltI* mutant indicate that the mutant cells experienced unfavorable hypertonic stress that promoted cell death. The genetic complementation of *gltI* and the exogenous addition of 1 mM glycine betaine to the *gltI* mutant recovered the wild-type cell shape and rescued the mutant strain from population collapse (Fig 8C and 8D).

## Discussion

The growth rate and survival of bacteria are dependent upon cellular osmolality during growth and incubation in culture media [24, 25]. To support cell turgidity and maintain an optimal growth rate, glutamate and other osmolytes are accumulated by uptake and/or synthesis during bacterial growth [25, 26]. In Gram-negative bacteria, glutamate is generally accumulated in response to hyperosmotic stress, allowing bacterial cells to grow in a wide range of osmolality [26, 27]. Regulation of cellular osmolality by the synthesis of glutamate has been relatively well studied in *E*. *coli* and *S*. *typhimurium* [2, 7, 9]. When a rapid increase in osmolality occurs in the organisms’ environment, the enteric bacteria take up potassium via osmolality-responsive transport systems [2, 7, 9]. Glutamate synthesis is then increased as a counter-ion to correspond to potassium uptake. This indicates that potassium-glutamate preferentially functions as an osmoprotectant upon exposure to high osmolality. Other organic osmolytes are then slowly taken up from the surroundings or synthesized *de novo* and accumulated [12, 20]. This fact may not be an exception in *B*. *glumae*. However, it is not known how glutamate uptake is controlled to maintain cellular osmolality and how critically important glutamate uptake from the environment is for the survival of *B*. *glumae*. We showed that glutamate concentration in the wild-type strain increased during growth as expected. However, when glutamate uptake was blocked in *B*. *glumae*, the glutamate accumulated very slowly, and the increase in the cellular level of glutamate was stalled. These results indicate that *B*. *glumae* takes up and accumulates glutamate from its environment if it is available rather than synthesizing glutamate *de novo* to maintain normal osmolality.

In this study, analysis of *glnA*, *gltB*, and *gdhA* gene expression in the *gltI* mutant showed that only the *gltB* gene was expressed at higher levels in the mutant than in the wild-type strain in the early growth stage, but not in stationary phase. These results indicate that the *de novo* synthesis of glutamate is independent of hypertonic stress due to the cell’s inability to take up glutamate from its environment and that *de novo* glutamate synthesis does not influence cellular glutamate concentration in the *gltI* mutant. Thus, the *gltI* mutant could not replenish cellular osmolality with sufficient glutamate by *de novo* synthesis. These results suggested that *B*. *glumae* adjusts cellular osmolality depending upon what is available in its environment. Glutamate is taken up from the culture medium as a nutrient and is primarily used to maintain cellular osmolality without a large contribution by the endogenous glutamate synthesis pathway in *B*. *glumae*. Aside from the fact that the *de novo* glutamate synthesis is negatively controlled by quorum sensing in *B*. *glumae* [22], we conclude that hyperosmolality due to loss of glutamate uptake does not activate the *de novo* synthesis of glutamate under crowded conditions.

Exogenous addition of 1 mM glycine betaine reversed the growth retardation and cell death that occurred in the *gltI* mutant, which was consistent with observations in other bacteria that growth retardation under high osmotic stress was relieved when glycine betaine was added to culture medium [28, 29]. Addition of glycine betaine to the culture medium had no effect on the growth rate of the wild-type strain, supporting the conclusion that the wild-type cultures were in steady-state growth when sufficient glutamate was present in the culture medium, and did not undergo osmotic stress without an exogenous supply of osmoprotectants. These results show that *B*. *glumae* uses glutamate as a primary solute for osmotic homeostasis in a glutamate-rich environment, such as LB medium.

This study clearly demonstrates that glutamate accumulation via the uptake of glutamate is a prerequisite for the efficient growth of *B*. *glumae* in nutrient-rich growth media, and for adaptation to osmotic stress conditions. The results also indicate that the *de novo* synthesis of glutamate does not substitute for glutamate uptake when *B*. *glumae* is exposed to osmotic challenges. Thus, the cellular glutamate level, cellular osmolality, and growth rate are correlated and connected in *B*. *glumae*.

## Supporting information

S1 Fig Cellular potassium ion levels in *B. glumae* strains. The levels of cellular potassium (mg/g) were measured in *B*. *glumae* strains cultured in LB medium for 6, 12, and 24 h. All samples were normalized by the weight of cells. Error bars represent the standard error (SE) ranges of three independent experiments.
(PDF)

S2 Fig Growth of the *B. glumae* strains with exogenous addition of glycine betaine. Growth of the *B*. *glumae* wild-type strain BGR1 (A), the *gltI* mutant (BGLT1) (B), and *gltI* mutant complemented with pGLT1 [BGLT1(pGLT1)] (C) with various concentrations of glycine betaine as a compatible solute in Luria-Bertani (LB) media. Error bars indicate the standard error ranges of triplicate experiments.
(PDF)

S1 Table Bacterial strains used in this study. (DOCX)

S2 Table Oligonucleotide primers used in this study. (DOCX)
