# Supplementary figures and images for: Glutamate uptake is important for osmoregulation and survival in the rice pathogen Burkholderia glumae
Source: PLoS One. 2018 Jan 2;13(1):e0190431. doi: 10.1371/journal.pone.0190431 (PMC5749808; doi:10.1371/journal.pone.0190431)

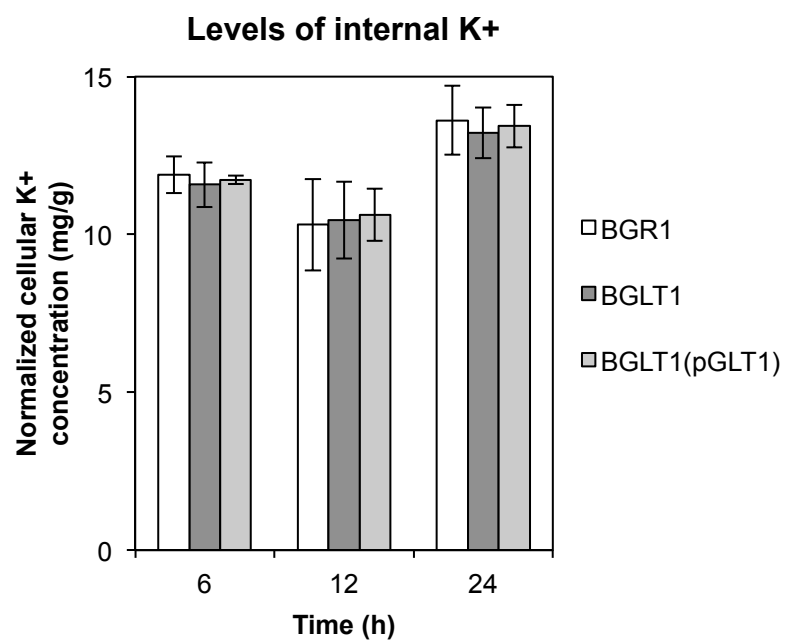

Supplement: S1 Fig — The levels of cellular potassium (mg/g) were measured in B. glumae strains cultured in LB medium for 6, 12, and 24 h. All samples were normalized by the weight of cells. Error bars represent the standard error (SE) ranges of three independent experiments. (PDF) [file pone.0190431.s001.pdf]

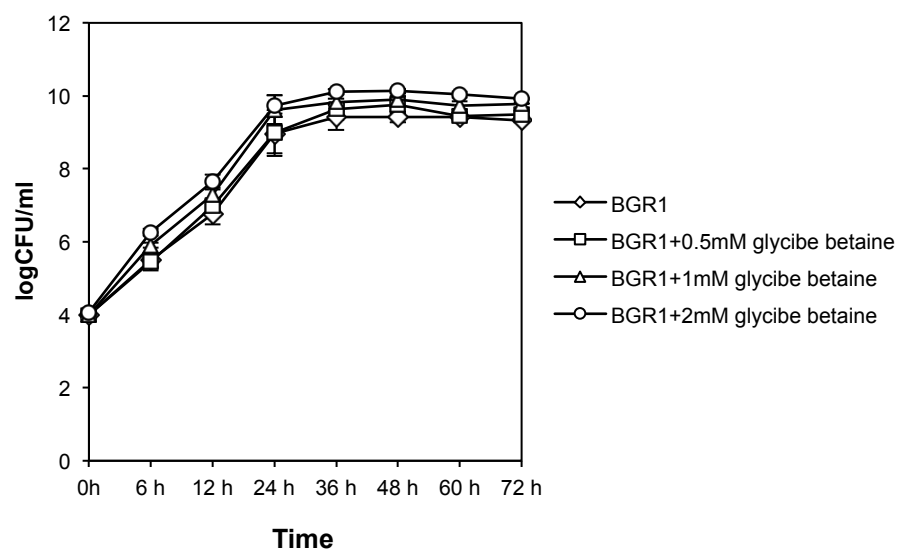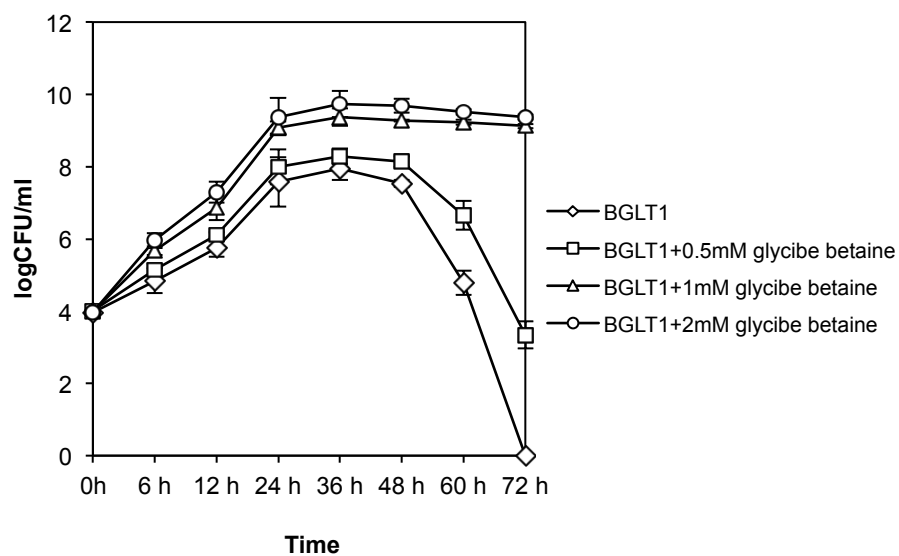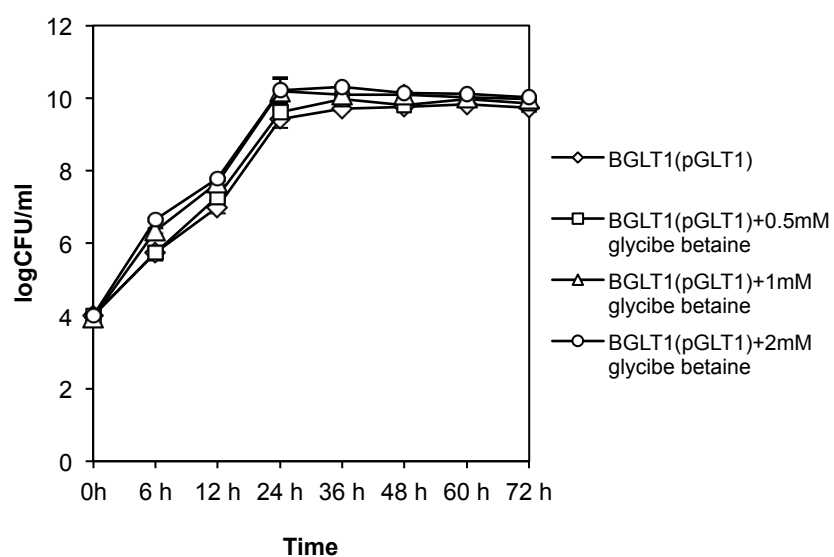

Supplement: S2 Fig — Growth of the B. glumae wild-type strain BGR1 (A), the gltI mutant (BGLT1) (B), and gltI mutant complemented with pGLT1 [BGLT1(pGLT1)] (C) with various concentrations of glycine betaine as a compatible solute in Luria-Bertani (LB) media. Error bars indicate the standard error ranges of triplicate experiments. (PDF) [file pone.0190431.s002.pdf]
