# Supplementary material for: Glutamate uptake is important for osmoregulation and survival in the rice pathogen Burkholderia glumae
Source: PLoS One. 2018 Jan 2;13(1):e0190431. doi: 10.1371/journal.pone.0190431 (PMC5749808; doi:10.1371/journal.pone.0190431)
Supplement: S1 Table — (DOCX) [file pone.0190431.s003.docx]

**S1 Table.**

| Strain or plasmid | Characteristics | Source or reference |
| --- | --- | --- |
| *Escherichia coli* | | |
| DH5α | F- Φ80d*lacZ* ΔM15 (*lacZYA*-*argF*) *U169* *recA1* *endA1* *hsdR17*(r_K_^+^m_K_^+^) *supE44* *thi*-*1* *gyrA* *relA1* | Gibco BRL |
| S17-1 | Tra^+^ *recA* Sp^R^ | [1] |
| *Burkholderia* *glumae* | | |
| BGR1 | Wild-type, Rif^R^ | [2] |
| BGLT1 | BGR1 *gltI*::Tn*5* | [3] |
| Plasmid | | |
| pLAFR3 | Tra^-^, Mob^+^, RK2 replicon, Tet^R^ | [4] |
| pGLT1 | 23.5 kb DNA fragment including the *gltI* gene from strain BGR1 cloned into pLAFR3 | [3] |

**References**

1. Simon R, Priefer U, Pühler A. A broad host range mobilization system for *in vivo* genetic engineering: transposon mutagenesis in Gram negative bacteria. Bio/Technology. 1983;1: 784–791.

2. Kim J, Kim JG, Kang Y, Jang JY, Jog GJ, Lim JY, et al*.* Quorum sensing and the LysR-type transcriptional activator ToxR regulate toxoflavin biosynthesis and transport in *Burkholderia glumae*. Mol Microbiol. 2004;54: 921–934.

3. Kang Y, Goo E, Kim J, Hwang I. Critical role of quorum sensing-dependent glutamate metabolism in homeostatic osmolality and outer membrane vesiculation in *Burkholderia glumae*. Sci Rep. 2017;7: 44195; doi: 10.1038/ srep44195

4. Staskawicz B, Dahlbeck D, Keen N, Napoli C. Molecular characterization of cloned avirulence genes from race 0 and race 1 of *Pseudomonas syringae* pv. *glycinea*. J Bact. 1987;169: 5789–5794.
