# Supplementary material for: Glutamate uptake is important for osmoregulation and survival in the rice pathogen Burkholderia glumae
Source: PLoS One. 2018 Jan 2;13(1):e0190431. doi: 10.1371/journal.pone.0190431 (PMC5749808; doi:10.1371/journal.pone.0190431)
Supplement: S2 Table — (DOCX) [file pone.0190431.s004.docx]

**S2 Table.**

| Primer name^a^ | Sequence (5′ to 3′) |
| --- | --- |
| GOGAT-L | CAAGGAGCTGAAGGACAACC |
| GOGAT-R | GAGCGTCTTGTTCTTGTTCG |
| GDH-L | GGATGTCGGTCAAGAATGC |
| GDH-R | TGGTTCATCGAGTAGGTGTCC |
| GS-L | GACACGTTCCAGGACATGC |
| GS-R | CCAGATCGACTGGTGAACG |

a. L, forward primer: R, reverse primer
